# Hypo-Osmotic Loading Induces Expression of IL-6 in Nucleus Pulposus Cells of the Intervertebral Disc Independent of TRPV4 and TRPM7

**DOI:** 10.3389/fphar.2020.00952

**Published:** 2020-07-01

**Authors:** Aleksandra Sadowska, Birsen Altinay, Wolfgang Hitzl, Stephen J. Ferguson, Karin Wuertz-Kozak

**Affiliations:** ^1^ Institute for Biomechanics, ETH Zurich, Zurich, Switzerland; ^2^ Research Office (Biostatistics), Paracelsus Medical University, Salzburg, Austria; ^3^ Department of Ophthalmology and Optometry, Paracelsus Medical University Salzburg, Salzburg, Austria; ^4^ Research Program Experimental Ophthalmology and Glaucoma Research, Paracelsus Medical University, Salzburg, Austria; ^5^ Tissue Regeneration & Mechanobiology Lab, Department of Biomedical Engineering, Rochester Institute of Technology (RIT), Rochester, NY, United States; ^6^ Spine Center, Schön Clinic Munich Harlaching, Academic Teaching Hospital and Spine Research Institute of the Salzburg Paracelsus Medical University, Munich, Germany

**Keywords:** intervertebral disc (IVD), osmolarity, osmosensing, transient receptor potential (TRP) channels, inflammation, degenerative disc disease (DDD), membrane receptor, low back pain

## Abstract

Painful intervertebral disc (IVD) degeneration is an age-related process characterized by reduced tissue osmolarity, increased catabolism of the extracellular matrix, and elevated levels of pro-inflammatory molecules. With the aging population and constantly rising treatment costs, it is of utmost importance to identify potential therapeutic targets and new pharmacological treatment strategies for low back pain. Transient receptor potential (TRP) channels are a family of Ca^2+^ permeable cell membrane receptors, which can be activated by multitude of stimuli and have recently emerged as contributors to joint disease, but were not investigated closer in the IVD. Based on the gene array screening, TRPC1, TRPM7, and TRPV4 were overall the most highly expressed TRP channels in bovine IVD cells. We demonstrated that TRPV4 gene expression was down-regulated in hypo-osmotic condition, whereas its Ca^2+^ flux increased. No significant differences in Ca^2+^ flux and gene expression were observed for TRPM7 between hypo- and iso-osmotic groups. Upon hypo-osmotic stimulation, we overall identified *via* RNA sequencing over 3,000 up- or down-regulated targets, from which we selected aggrecan, ADAMTS9, and IL-6 and investigated whether their altered gene expression is mediated through either the TRPV4 or TRPM7 channel, using specific activators and inhibitors (GSK1016790A/GSK2193874 for TRPV4 and Naltriben/NS8593 for TRPM7). GSK1016790A induced the expression of IL-6 under iso-osmotic condition, alike to hypo-osmotic stimulation alone, indicating that this effect might be TRPV4-mediated. However, using the TRPV4 blocker GSK2193874 failed to prevent the increase of IL-6 under hypo-osmotic condition. A treatment with TRPM7-activator did not cause significant changes in the gene expression of tested targets. In conclusion, while TRPV4 and TRPM7 are likely involved in osmosensing in the IVD, neither of them mediates hypo-osmotically-induced gene expression changes of aggrecan, ADAMTS9, and IL-6.

## Introduction

The IVD is a mechanically loaded structure composed of two main tissues – the centrally located and highly hydrated nucleus pulposus (NP), which is surrounded by circular lamellar rings known as annulus fibrosus (AF) (Cassinelli et al., 2001). In a healthy state, the NP contains a small population of cells embedded in a loose matrix of collagen (COL) type II and proteoglycans (PGs), which enable the NP’s high water content (60–99%) and subsequent osmotic pressure (Cassinelli et al., 2001; Roughley et al., 2002). In contrast, the AF is mostly composed of COL type I, which gives the tissue the ability to retain tensile stresses and hydrostatic pressure generated from NP (Cassinelli et al., 2001). Age- and/or degeneration-induced changes in proteoglycan content lead to a drop in osmotic concentration from ~400 mOsm/L in a healthy state to ~300 mOsm/L in a degenerated IVD (Wuertz et al., 2007). Other common occurrences observed with IVD degeneration include, among others, a shift in extracellular matrix (ECM) metabolism towards catabolism and up-regulated expression of matrix metalloproteinase (MMPs) or disintegrin and metalloproteinase with thrombospondin motifs (ADAMTS) (Le Maitre et al., 2004). Correspondingly, biochemical and structural changes to the IVD affect its mechanical function and can promote inflammation. Inflammation in the IVD can be characterized by an increased expression of pro-inflammatory molecules such as interleukin (IL)-6, IL-1β, and tumor necrosis factor alpha (TNF-α), and contributes to the development of degenerative disc disease (DDD) and low back pain (LBP) (Molinos et al., 2015). Discogenic LBP not only reduces the patient’s quality of life, but also creates a high economic burden on the individuals and the society (Wieser et al., 2011). Current treatment strategies are mostly limited to oral pain medication, physiotherapy, lumbar epidural steroid injections, and surgeries (or a combination thereof), all of which are rather reactionary than preventive measures and reported efficacy varies between publications (Koc et al., 2009; Ulger et al., 2018). Hence, there is a clear need to not only develop new treatment strategies, but also to identify specific therapeutic targets, which mediate and/or promote homeostatic or inflammatory processes in the IVD.

Transient Potential Receptor (TRP) channels are a superfamily of multimodal cation membrane receptors and have recently emerged as potential contributors to IVD and joint diseases as well as to discogenic pain. They can be activated by multiple stimuli, including mechanical and osmotic stress (e.g. TRP canonical [C] and TRP vanilloid [V] subfamilies) and function as cellular sensors (Krupkova et al., 2017). TRP channels have previously been shown to be well expressed in the human IVD; importantly the gene and protein expression of some TRP channels has been reported to be degeneration-, pain intensity- and/or pain chronicity-dependent (Sadowska et al., 2019). A past study on IVD degeneration reported increased TRPV4 gene expression with a decrease in osmolarity (~400 mOsm/L *vs.* ~300 mOsm/L and below) and suggested that TRPV4 signaling may mediate increased expression of IL-1β and IL-6 (Walter et al., 2016). TRPM3 and TRPM7 channels, which so far were sparsely investigated in the IVD, are implicated in sensing of osmotic changes and mediation of osmolarity-induced cell volume changes in human renal cells and salivary glands (Grimm et al., 2003; Harteneck and Reiter, 2007). Furthermore, hypo-osmotic stretch was also shown to mechanically activate TRPC5 and TRPC6 channels in the central and peripheral nervous system and in renal cells (Gomis et al., 2008; Wilson and Dryer, 2014). Hence, TRP channels constitute a promising target for the investigation of IVD degeneration and accompanying reduced tissue osmolarity. Thus far, it is unclear which TRP channels may function as osmosensors in the IVD and whether they mediate catabolic and inflammatory changes in the response to hypo-osmotic stress. Therefore, the goal of this study was to

1. Identify the most prominently expressed TRP channels in bovine caudal NP and AF cells by gene array screening.
2. Investigate how changes in osmolarity affect the expression and activity of the identified TRP channels.
3. Identify pro-inflammatory and ECM targets with altered gene expression, due to short- and long-term exposure to reduced osmolarity, and to determine whether these changes are TRP channel-mediated (= main objective).

## Materials and Methods

### Bovine Nucleus Pulposus Cell Isolation and Culture

Due to the limited accessibility of healthy human IVD tissue, healthy bovine caudal discs were used in this study. Bovine caudal discs are considered to be a suitable model for the study of the human lumbar disc (especially that of a young adult), due to their biological and biomechanical similarity to the human IVD (Demers et al., 2004). All experiments were conducted on n = 3–7 biological replicates, as indicated in each results section.

Bovine tails from 18- to 24-month-old male and female animals were obtained from a local slaughterhouse. Bovine nucleus pulposus (NP) and annulus fibrosus (AF) cells were isolated as previously described (Wuertz et al., 2007). Within 1–2 h after the slaughter, caudal IVDs were dissected under sterile conditions, where NP, AF, and the transition zone (TZ) were separated from each other using either a 8, 6, or 3 mm biopsy tool and a blade. For each animal, the top eight IVD sections were used. Collected AF or NP tissue was pooled together from each animal, whereas remaining TZ tissue was discarded. The tissue was cut into fine pieces and digested overnight at 37°C, 5% CO_2_ in a solution composed of 3 mg/ml Collagenase NB 4 (#S1745401, Nordmark Biochemicals, Germany), 2 mg/ml Dispase II (#2845300, Roche Diagnostics USA), and 3% antibiotic-antimycotic (A/A, #15240-062, Gibco Life Technologies, Switzerland) dissolved in 100 ml of sterile phosphate buffered saline (PBS, #09-8912-100, Medicago Sweden). On the next day, the tissue digest was filtered using cell strainers (70 μm, #542070, Greiner Bio-One, Switzerland) and centrifuged at 1,000 rpm for 20 min at the room temperature (RT), with three washing steps (1× PBS, 2× cell culture media) in between. Cells from different donors were not pooled together, but used separately as biological replicates.

For the TRP screening experiment, cells were collected either directly after isolation or sub-cultured in Dulbecco’s Modified Eagle Medium: Nutrient Mixture F-12 (DMEM/F12, #11320033 Gibco, Switzerland; 300 mOsm, “hypo-osmotic”) supplemented with 10% fetal calf serum (FCS, #F7524, Sigma-Aldrich, Switzerland) and 1% A/A until passage (P) 2 and collected for the analysis afterwards.

For the remaining sub-culturing, cells were seeded in DMEM/F12 adjusted to ~400 mOsm (“iso-osmotic” media) using sucrose (#57903, Sigma-Aldrich, Switzerland) and supplemented with 0.1% Ampicillin (#A6352.0025, PanReac AppliChem Switzerland) and 10% FCS. Osmolarity was measured using a freezing-point osmometer (Osmomat 030, Gonotec, Germany). Serum free hypo- and iso-osmotic media was used for cell treatment experiments. Additionally, to ensure consistency throughout treatments, the osmolarity of PBS used for cell washing during cell splitting or in assays, was correspondingly adjusted to match the treatment osmolarity.

### Treatments

Bovine NP cells (P2) were seeded in 6-well plates (0.2 × 10^6^ cells/well; gene expression analysis/ELISA), 24-well plates (0.65 × 10^5^ cells/well, for MTT assay), or 96-well plates (0.1 × 10^5^ cells/well, for [Ca2+] imaging) and cultured for 24 h. On the following day, cells were serum-starved for up to 3 h and treated (**Table 1**) for assay-dependent durations in serum free, osmotically adjusted media with channel specific agonist, antagonist, or DMSO (negative control): gene expression analysis/ELISA = 24 h; [Ca2+] imaging = 15–30 min; MTT assay = 15 min to 24 h. Afterwards, cells were directly used for MTT assay/qPCR. For ELISA, cell culture media was collected before and after the treatment.

**Table 1:** Chemical compounds and concentrations.

Compound	Catalog number, Manufacturer	Function	MTT concentration [µM]	[Ca2+] imaging concentration [µM]	Cell treatmentconcentration [µM]
GSK1016790A	#17289, Cayman Chemical Company, CH	TRPV4 agonist	0.005, 0.02, 0.025, 0.04, 0.05	0.002, 0.02, 0.04	0.05
GSK2193874	# 1336960134, Sigma-Aldrich, CH	TRPV4 antagonist	0.1, 0.5, 1, 10	0.25, 0.5	0.5
Naltriben methanesulfonate hydrate	#N156, Sigma-Aldrich, CH	TRPM7 agonist	5, 15, 20, 25, 50, 200, 300, 400, 500, 600	50, 100, 200	25
NS8593 hydrochloride	#N2538, Sigma-Aldrich, CH	TRPM7 antagonist	150, 200, 250, 300, 350, 400	80, 160	–

### RNA Sequencing and Data Analysis

Twelve RNA samples were submitted to the Functional Genomics Center in Zurich (FGCZ) for RNA sequencing. The quality of the RNA was determined with a Fragment Analyzer standard sensitivity RNA measurement (SS RNA kit [15 nt], Agilent, Waldbronn, Germany). The measured concentrations (>75 ng/µl) and RIN (>9.9) values qualified for a Poly-A enrichment strategy in order to generate the sequencing libraries applying the TruSeq mRNA Stranded Library Prep Kit (Illumina, Inc, California, USA). After Poly-A selection using Oligo-dT beads the mRNA was reverse-transcribed into cDNA. The cDNA was fragmented, end-repaired, and polyadenylated before ligation of TruSeq UD Indices (IDT, Coralville, Iowa, USA). The quality and quantity of the amplified sequencing libraries were validated using a Fragment Analyzer SS NGS Fragment Kit (1–6,000 bp) (Agilent, Waldbronn, Germany). The equimolar pool of 12 samples was spiked into a NovaSeq6000 run targeting 200M reads on a S1 FlowCell (Novaseq S1 Reagent Kit, 100 cycles, Illumina, Inc, California, USA). The Bcl files were demultiplexed using Illumina`s bcltofastq software allowing for one mismatch in each barcode.

The RNA-seq data analysis consisted of the following steps: The raw reads were first cleaned by removing adapter sequences, trimming low quality ends, and filtering reads with low quality (phred quality <20) using Trimmomatic (Version 0.36) (Bolger et al., 2014). The read alignment was done with STAR (v2.7.0e) (Dobin et al., 2013). As reference we used the Ensembl genome build UMD_v3.1 with the gene annotations downloaded on 2018-05-30 from Ensembl (release 92). The STAR alignment options were “–outFilterType BySJout –outFilterMatchNmin 30 –outFilterMismatchNmax 10 –outFilterMismatchNoverLmax 0.05 –alignSJDBoverhangMin 1 –alignSJoverhangMin 8 –alignIntronMax 100000 –alignMatesGapMax 100000 –outFilterMultimapNmax 50”. The quantification of transcript level expression was carried out using Kallisto (Version 0.44) (Bray et al., 2016). To detect differentially expressed genes we applied a count based negative binomial model implemented in the software package EdgeR (R version: 3.6.0, EdgeR version: 3.26.8) (Robinson et al., 2010). The differential expression was assessed using an exact test adapted for over-dispersed data. Genes showing altered expression with adjusted (Benjamini and Hochberg method) p-value <0.05 were considered differentially expressed.

### Gene Expression Analysis

RNA was extracted using the RNeasy Mini Kit (#74106, Qiagen, Switzerland) following the manufacturer’s protocol. One microgram of RNA was used to synthesize cDNA in a total volume of 30 µl, using a reverse transcription kit (#4374966, Applied Biosystems, USA).

The expression of TRP channels in bovine NP and AF cells was screened using custom TaqMan Array Fast Plates (#4413261, Thermo Fisher, Switzerland) following the manufacturer’s recommendations. In the first step, obtained cDNA was amplified using TaqMan PreAmp Master Mix (2×) (#4391128, Thermo Fisher, Switzerland) and Custom TaqMan PreAmp Pools (Thermo Fisher, Switzerland). Then, amplified cDNA (mixed with RNAse-free water) was combined 1:1 with the TaqMan Fast Universal PCR Master Mix (2×) (#4352042, Thermo Fisher, Switzerland) and added to target pre-coated (TRP channels, **Table 2**) 96-well plates (10 µl per well). The gene expression was measured using real-time qPCR (CFX96 Touch™ Detection System, Biorad).

**Table 2:** TaqMan primers list.

Gene symbol	Gene name	TaqMan Primer Assay ID
ACAN	Aggrecan	Bt03212186_m1
ADAMTs4	Adam Metallopeptidase With Thrombospondin Type 1 Motif 4	Bt03224697_m1
ADAMTs9	Adam Metallopeptidase With Thrombospondin Type 1 Motif 9	Bt04295942_m1
COL2	Collagen Type II Alpha 1 Chain	Bt03251861_m1
CHOP (a.k.a. DDIT3)	Dna Damage Inducible Transcript 3	Bt03251320_g1
HAS2	Hyaluronic Acid Synthase 2	Bt03212695_g1
HAS3	Hyaluronic Acid Synthase 3	Bt04298491_m1
GRP78 (a.k.a HSPA5)	Heat Shock Protein Family A (Hsp70) Member 5	Bt03244880_m1
COX2 (a.k.a. PTGS2)	Prostaglandin-Endoperoxide Synthase	Bt03214492_m1
IL-6	Interleukin 6	Bt03211904_m1
MMP3	Matrix Metallopeptidase 3	Bt04259497_m1
TNFRSF21	Tumor Necrosis Factor Receptor Superfamily Member 21	Bt03250597_m1
TRPC1	Transient Receptor Potential Cation Channel, Subfamily C, Member 1	Bt03214647_m1
TRPC2	Transient Receptor Potential Cation Channel, Subfamily C, Member 2	Bt03817472_m1
TRPC3	Transient Receptor Potential Cation Channel, Subfamily C, Member 3	Bt03258742_m1
TRPC4	Transient Receptor Potential Cation Channel, Subfamily C, Member 4	Bt03214662_m1
TRPC5	Transient Receptor Potential Cation Channel, Subfamily C, Member 5	Bt04301428_m1
TRPC6	Transient Receptor Potential Cation Channel, Subfamily C, Member 6	Bt04301412_m1
TRPC7	Transient Receptor Potential Cation Channel, Subfamily C, Member 7	Bt04297646_m1
TRPM3	Transient Receptor Potential Cation Channel, Subfamily M, Member 3	Bt03243121_m1
TRPM7	Transient Receptor Potential Cation Channel, Subfamily M, Member 7	Bt04290223_m1
TRPM8	Transient Receptor Potential Cation Channel, Subfamily M, Member 8	Bt04288753_m1
TRPV2	Transient Receptor Potential Cation Channel, Subfamily V, Member 2	Bt03210789_m1
TRPV3	Transient Receptor Potential Cation Channel, Subfamily V, Member 3	Bt03262740_m1
TRPV4	Transient Receptor Potential Cation Channel, Subfamily V, Member 4	Bt03649002_m1
TRPV6	Transient Receptor Potential Cation Channel, Subfamily V, Member 6	Bt04290617_m1
YWHAZ	Tyrosine 3-Monooxygenase/Tryptophan 5-Monooxygenase Activation Protein, Zeta Polypeptide	Bt01122444_g1

For the remaining gene expression analysis, TaqMan primers (**Table 2**, Thermo Fisher, Switzerland) were used according to the manufacturer’s protocol. In short, 5 µl of TaqMan Fast Universal PCR Master Mix (2×) was mixed with 0.5 µl of TaqMan primers and 10 ng cDNA (combined with RNAse-free water for 4.5 µl total volume), and quantified using the real-time qPCR. Data were analyzed either as 2^−ΔCt^ values relative to the housekeeping gene (YWHAZ) or as fold change 2^−ΔΔCt^ values normalized to YWHAZ and to a control. YWHAZ was chosen as the housekeeping gene based on its stability in preliminary testing.

### [Ca2+] Imaging

To investigate the activity of a TRP channel in hypo- and iso-osmotic environments, Fura-2 QBT™ Calcium Kit (#R8197, Molecular Devices, UK) was used following the manufacturer’s protocol to detect intracellular calcium changes. In short, bovine NP cells (n = 3, P2, 0.1 × 10^5^ cells/well) were seeded in a 96-well plate in iso-osmotic media supplemented with 10% FCS and 0.1% Ampicillin. On the next day, media was changed to osmotically adjusted (300 or 400 mOsm) phenol-red free media (DMEM/F-12, #11039021, Gibco, Switzerland) supplemented with 0.1% Ampicillin. After around 2–3 h, an equal volume of Fura-2 Loading Buffer was added to each well, followed by an incubation for 1 h at 37°C, 5% CO_2_. Next, cells were treated with either TRPV4 antagonist, TRPM7 antagonist, or DMSO (negative control, 0.01 or 1%) for 15 min at the RT (**Table 1**). Each compound was dissolved in DMSO at 10 mM stock concentration and stored in aliquots at −20°C and was freshly diluted into various working concentrations right before the use (**Table 1**). Calcium response was measured using a microplate reader (Tecan, Infinite M200 PRO). First, a baseline over seven cycles was recorded at an excitation wavelength of 340 nm for the bound calcium and 380 nm for the unbound calcium. At the eighth cycle, cells were treated with different concentrations of agonists (**Table 1**), and the measurement was continued until the 30th cycle, which corresponds to approximately 20 min of the total measurement time. Calcium response was analyzed as a ratio of the bound calcium to the unbound calcium (340 nm/380 nm), normalized to the baseline.

### MTT Assay

In order to test whether applied treatments influenced cell metabolic activity, MTT assay (3-[4,5-dimethylthiazol-2-yl]-2,5-diphenyl tetrazolium bromide, #M5655, Sigma-Aldrich, Switzerland) was used. Cells were cultured and treated as described beforehand (*Treatments* and **Table 1**). As a negative control, cells were treated with 70% methanol (#M5655, Sigma-Aldrich, Switzerland) for 30 min at 37°C, 5% CO_2_. Following treatments, the supernatants were aspirated, cells were washed with PBS and incubated with freshly prepared MTT solution (0.5 mg/ml, dissolved in media) for 1 h at 37°C, 5% CO_2_. Thereupon, MTT was discarded and 200 µl DMSO was added to each well and incubated on a shaker for 5 min at RT. Subsequently, the lysates were transferred to 96-well plates in duplicates and the absorbance of formazan was measured at 565 nm using a microplate reader (Tecan, Infinite M200 PRO). Metabolic activity was calculated relative to the untreated control (set at 100% cell viability).

### Immunocytochemistry (ICC)

Bovine NP cells in P2 were seeded into the chambered cover glass (Nunc Lab-Tek, #155380 or #154461, Thermo Fisher, Switzerland). Media was replaced to hypo- or iso-osmotic, FCS free media and cells were incubated for 24 h. Afterwards, cells were briefly washed 3× with PBS, fixed with ice cold 100% methanol (10 min at −20°C, #34885, Sigma-Aldrich, Switzerland), and blocked with 5% normal goat serum (#005-000-121, Jackson ImmunoResearch, PA, USA) in PBS for 1 h at the RT. Next, cells were incubated with the primary antibody (anti-TRPM7, #ACC-047 or anti-TRPV4, #ACC-034, Alomone, Israel, 1:500 in 1% goat serum in PBS) in the dark at 4°C overnight. On the next day, cells were first washed with PBS (3 × 10 min on a rocker) and then incubated with the secondary antibody (Cy2 anti-rabbit IgG, #111225144, Jackson ImmunoResearch, PA, USA; 1:200 in 1% normal goat serum) for 1 h at the RT. Next, cells were again washed with PBS (3 × 10 min on a rocker) and 1–2 drops of the Antifade Mounting Medium with DAPI (VECTASHIELD, #H-1200, Switzerland) were added shortly before imaging. Cells were imaged with a fluorescence microscope (20×, Olympus IX51 or 100×, Delta Vision System). For each primary antibody, the same imaging parameters (exposure time and magnification) were used. As a negative control, cells were incubated without primary antibody.

### Enzyme-Linked Immunosorbent Assay (ELISA)

To quantify the release of IL-6 from treated IVD cells, the cell culture media was collected before and after treatment, and analyzed with IL-6 ELISA kit following the manufacturer’s protocol (IL-6, #ESS0029, Thermo Fisher, Switzerland). Briefly, 96-well plates were coated with coating antibody (1:100) overnight. On the next day, wells were blocked (ELISA Blocking Buffer, #N502, Thermo Fisher, Switzerland) for 1 h at RT and afterwards loaded with samples or protein standard, and incubated for 1 h at RT. Next, wells were washed (ELISA Wash Buffer 1×, #N503, Thermo Fisher, Switzerland), incubated with the detection antibody (1:100) for 1 h at RT, followed by washing and 1 h incubation with streptavidin-horseradish peroxidase (HRP) at RT. Next, wells were washed again and substrate solution was added to each well. After 20 min of incubation in the dark, stop solution was added to each well, the absorbance was directly measured (Tecan, Infinite M200 PRO) at 450 nm (with subtracted 550 nm absorbance), and IL-6 concentrations were calculated based on the standard curve.

### Statistical Analysis

All data were checked for consistency and screened for outliers. For the gene array, dependent bootstrap t-test based on 7000 Monte Carlo samples was used due to the small sample sizes. In addition, classical dependent t-tests and nonparametric tests (Wilcoxon Signed test) and Quantile Sign test was used. For the calcium assay, continuous variables were also tested for normality. Generalized estimation equation models based on Gamma distributions were used to analyze data. The robust estimator for the covariance matrix was used and a full factorial model was set up. Finally, LSD tests were used to compare means pairwise. For the MTT and gene data, One-way ANOVA followed by Tukey’s or Dunnett’s multiple comparisons test was used to test means among different groups. All reported tests were two-sided, p-values ≤0.05 were considered statistically significant and all error bars present SEM. All statistical analyses in this report were performed by use of NCSS (NCSS 10, NCSS, LLC. Kaysville, UT) and PASW 24 (IBM SPSS Statistics for Windows, Version 21.0., Armonk, NY) or GraphPad Prism version 8.2.0 for Windows (GraphPad Software, La Jolla California USA, www.graphpad.com).

## Results

### TRP Channels mRNA Expression in Bovine NP and AF Cells

**Figure 1:**
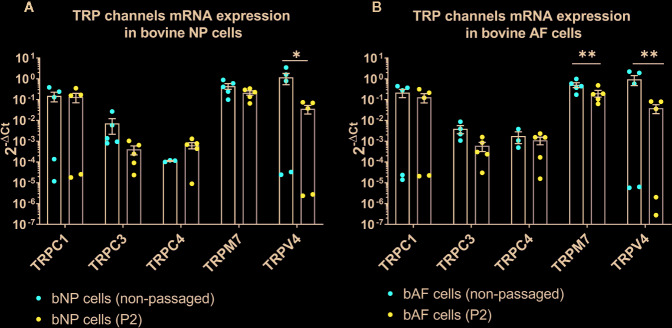
The mRNA expression of TRP channels in **(A)** bovine nucleus pulposus (bNP) and **(B)** annulus fibrosus (bAF) cells obtained directly after cell isolation (non-passaged) or cells passaged until passage 2 (P2). Graphs show 2^−ΔCt^ values relative to YWHAZ (mean ± SEM, n = 5). Asterisks indicate statistical significance (*p < 0.05, **p < 0.01) between non-passaged and passaged cells.

The mRNA expression of 14 TRP channels (TRPC1-C7, TRPM3, TRPM7, TRPM8, TRPV2-V2, and TRPV6) was tested in bovine NP and AF cells obtained directly from tissue digest or passaged cells collected at P2. In the initial test, a set of three donors was used with Taqman Array Plates. **Table 3** presents the detectability of all tested TRP channels. Five out of 14 TRP targets, namely TRPC1, TRPC3, TRPC4, TRPM7, and TRPV4, were selected for further gene analysis on additional two donors. This decision was made based on either highest overall expression (e.g. TRPC1, TRPM7, and TRPV4), novelty (TRPC1, TRPC3, TRPC4), or research relevance in the IVD or other tissue types. The detectability of expression and relative mRNA expression levels from all five donors are presented in **Table 4** and **Figure 1**, respectively. The gene expression tended to be generally higher in non-passaged cells as compared to passaged cells (**Figures 1A, B** **)**. This difference was statistically significant (p < 0.05) for TRPV4 channel in NP cells (**Figure 1A**) as well as TRPC3, TRPM7, and TRPV4 channels in AF cells (**Figure 1B**). For the reason of overall high expression and the availability of channel specific agonists and antagonists, TRPM7 and TRPV4 were chosen out of the five pre-selected TRP channels for the subsequent experiments on the NP cells. Due to their relevance to osmotic changes in the IVD, only NP cells were included in the further experiments.

**Table 3:** Primary screening: number of donors (min. n = 0; max. n = 3), in which the mRNA expression of all available TRP channel was detectable using TaqMan gene array.

Target	NP digest	NP cells P2	AF digest	AF cells P2
TRPC1	3/3	3/3	3/3	3/3
TRPC2	1/3	1/3	2/3	–
TRPC3	3/3	3/3	2/3	3/3
TRPC4	1/3	3/3	1/3	3/3
TRPC5	2/3	–	1/3	–
TRPC6	1/3	–	3/3	1/3
TRPC7	–	1/3	–	2/3
TRPM3	3/3	3/3	2/3	3/3
TRPM7	3/3	3/3	3/3	3/3
TRPM8	1/3	3/3	–	3/3
TRPV2	3/3	3/3	3/3	3/3
TRPV3	1/3	–	1/3	1/3
TRPV4	3/3	3/3	3/3	3/3
TRPV6	3/3	3/3	3/3	3/3

“-”: not expressed in any of the tested samples.

**Table 4:** Secondary screening: number of donors (min. n = 0; max. n = 5), in which the mRNA expression of all selected TRP channels was detectable using TaqMan gene array and qPCR.

Target	NP digest	NP cells P2	AF digest	AF cells P2
TRPC1	5/5	5/5	5/5	5/5
TRPC3	5/5	5/5	4/5	3/5
TRPC4	3/5	5/5	3/5	5/5
TRPM7	5/5	5/5	5/5	5/5
TRPV4	5/5	5/5	5/5	5/5

### Effects of Hypo-Osmotic Stimulation on TRPV4 and TRPM7 mRNA and Protein Expression

**Figure 2:**
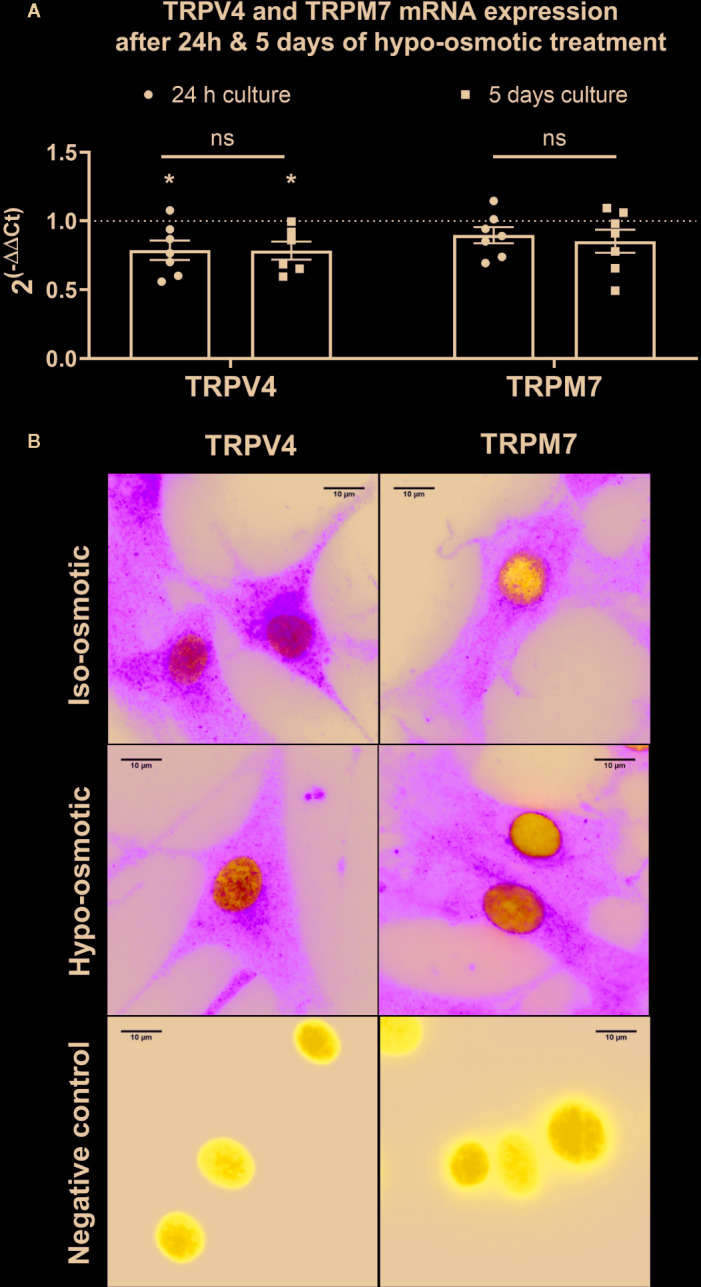
The mRNA expression of **(A)** TRPV4 (*left*) and TRPM7 (*right*) channels in bovine nucleus pulposus (NP) cells. Graphs show 2^−ΔΔCt^ values normalized to the iso-osmotic control (mean ± SEM, n = 6-7). Asterisks indicates statistical significance (*p < 0.05) between iso- and hypo-osmotic conditions, bars compare the difference between 24 h and 5-day long treatments (*ns*, no statistical difference defined as p > 0.05). **(B)** Protein expression of TRPV4 (left top and middle) and TRPM7 (right top and bottom) after 24 h of culture in either iso- (top) or hypo-osmotic media (middle). For the negative control (bottom left and right) cells were incubated without primary antibody. TRPV4/M7 are stained green, nuclei were counterstained with DAPI (blue). Scale bar is 10 µm, n = 3.

In the next step, TRPV4 and TRPM7 expression on the gene and protein level in both hypo- and iso-osmotic conditions was tested in bovine NP cells. For gene expression, cells were collected after either 24 h or 5 days of osmotic treatment. TRPV4 was slightly, but significantly, down-regulated in hypo-osmotic condition as compared to the iso-osmotic group at both collection points *versus* iso-osmotic groups (fold change 24 h: mean 0.79, min. 0.56, max. 1.07, p = 0.03 and fold change day 5: mean 0.79, min. 0.56, max. 1, p = 0.04, **Figure 2A** left). The expression of TRPM7 was unchanged at both collection points (fold change 24 h: mean 0.9, min. 0.69, max. 1.14, p = 0.45 and fold change day 5: mean 0.85, min. 0.49, max. 1.1. p = 0.21, **Figure 2A** right). Correspondingly, on the protein level, the two channels were abundantly and steadily expressed in both osmotic conditions (**Figure 2B**: TRPV4 [left top and bottom] and TRPM7 [right top and bottom]) after 24 h of culture. While both channels were present in the cell membrane, TRPM7 additionally showed a clear localization around the nuclear envelope.

### Osmotic Regulation of TRPV4 and TRPM7 Channels Activity

**Figure 3:**
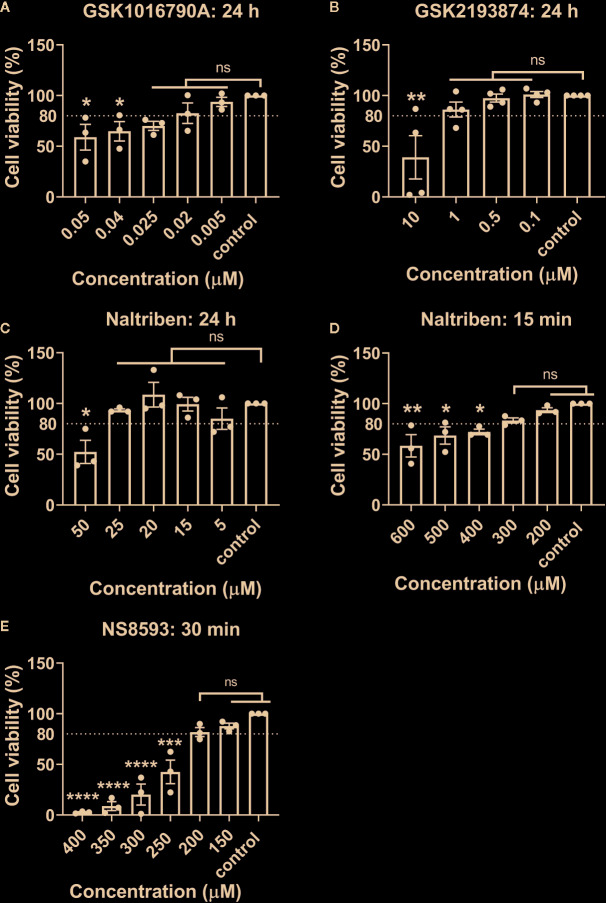
MTT results of bovine NP cells after different incubation periods with **(A)** GSK1016790A (TRPV4 activator), **(B)** GSK2193874 (TRPV4 blocker), **(C, D)** Naltriben methanesulfonate hydrate (TRPM7 activator), and **(E)** NS8593 hydrochloride (TRPM7 blocker). Graphs show percentage of viable cells (mean ± SEM, bNP P2, n = 3–4). Asterisks indicates statistical significance compared to the untreated control (*p < 0.05, *p < 0.01, ***p < 0.001, ****p < 0.0001, *ns*, no statistical difference defined at p > 0.05).

**Figure 4:**
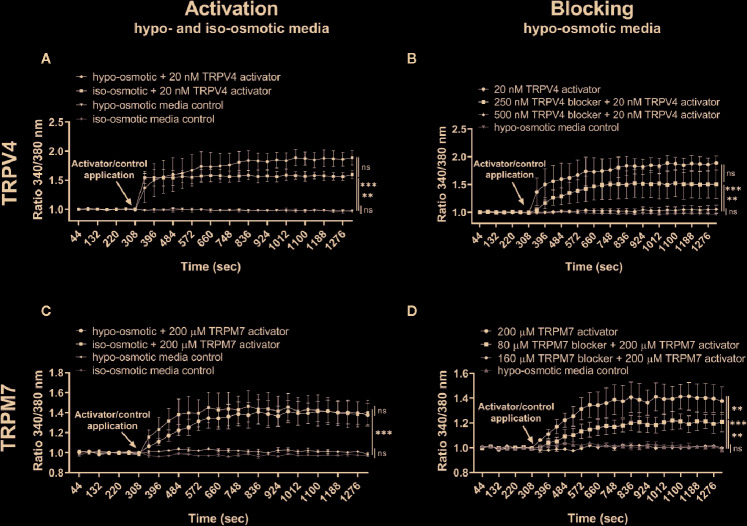
Ca^2+^ flux in NP cells following the application of **(A)** 20 nM GSK1016790A (TRPV4 activator) alone, **(B)** 250 or 500 nM GSK2193874 (TRPV4 blocker) and/or 20 nM GSK1016790A (TRPV4 activator), **(C)** 200 µM Naltriben (TRPM7 activator), and **(D)** 80 or 160 µM NS8593 hydrochloride (TRPM7 blocker) and/or 200 µM Naltriben (TRPM7 activator). Graphs present the ratio of the bound calcium to the unbound calcium (340 nm/380 nm) normalized to the baseline over the measurement time (mean ± SEM, bNP P2, n = 3). After the baseline measurement, empty (control) or compound-supplemented (treatment) media was added (indicated by an arrow on a graph) and the measurement was continued for up to around 15 min more. Asterisks indicate statistical significance (**p < 0.01, ***p < 0.001, *ns*, no statistical difference defined as p > 0.05) as measured on the last measurement cycle.

Channel-specific activators and blockers of TRPV4 and TRPM7 (**Table 1**) were employed to test whether their activity differs between iso- and hypo-osmotic conditions. To ensure non-toxicity of channel activators and blockers, their dose-dependent effect on the metabolic activity of bovine NP cells was tested using MTT assay (**Figure 3**). Next, the activity of a channel was measured using a calcium flux assay (n = 3). Based on the MTT results, three highest, non-toxic doses of an activator were applied to untreated bovine NP cells to establish the effective concentration of an agonist necessary to activate TRPV4 or TRPM7 channel in bovine NP cells (**Supplementary Figures 1** and **2**). In the final step, a selected concentration of an activator was applied to cells cultured in either iso- or hypo-osmotic media and the response was measured for up to 15 min from the activator injection time (**Figures 4A, C** **)**. To ensure the specificity of an activator, respective channel blockers were applied 15 min before the measurement (**Figures 4B, D** **)**. TRPV4 mediated calcium flux was higher in cells cultured in hypo-osmotic media as compared to iso-osmotic media (**Figure 4A**, p = 0.1), and the effect became significantly different (p = 0.002) with the increased dose of the GSK1016790A (40 nM, **Supplementary Figure 3** **).** There was no statistical difference in the response to TRPM7 activator between iso- or hypo-osmotic treated cells, and the calcium flux was similarly high in both conditions (**Figure 4C**, p > 0.05).

### Effects of Hypo-Osmotic Stimulation on Genome-Wide Expression Changes in NP Cells

Bovine NP cells were cultured in osmotically adjusted media for either 24 h or 5 days and were thereafter collected for the RNA sequencing or qPCR analysis. Overall, there were 38 differently up- or down-regulated targets after 24 h of hypo- *versus* iso-osmotic stimulation, and 3,062 differently up- or down-regulated targets after 5 days of hypo- *versus* iso-osmotic culture, which met the criterion of false discover rate (FDR) <0.05 and p-value <0.05. Out of those regulated at the 24 h time point, 12 targets were up-regulated at least 2 fold and 22 targets were down-regulated by at least 0.5 fold. In the 5 days group, 188 out of 3,062 targets were up-regulated by at least 2 fold and 419 targets were down-regulated by at least 0.5 fold. **Supplementary Tables 1** and **2** present the results of gene set enrichment analysis (GSEA) organized by a function of up- or down-regulated genes for 24 h and 5 days of osmotic culture, respectively. **Tables 5** and **6** present selected significantly up- and down-regulated targets for 24 h and 5 days long hypo- *versus* iso-osmotic culture, respectively (organized by fold change). Based on these results, 12 targets (ACAN, ADAMTS9, IL-6, ADAMTS4, COL2, COX2, MMP3, TNFRSF21, CHOP10, HAS2, HAS3, and GRP78) were selected for qPCR testing due to differential gene regulation, novelty, or relevance to inflammation and IVD degeneration or discogenic pain.

**Table 5:** RNA sequencing: top up- and down-regulated genes in bovine NP cells with FDR < 0.05 and p-value < 0.05 for 24 h of hypo-osmotic *versus* iso-osmotic treatment.

Top up-regulated targets		Top down-regulated targets	
Gene name	Fold change	Gene name	Fold change
BNIP3	4.30	NOV	0.47
CCL5	3.72	SLC44A1	0.47
SLC2A3	3.03	ERICH5	0.46
IL6	2.83	NUPR1	0.44
PTGS2	2.68	ARHGAP24	0.43
DDIT4	2.63	SESN3	0.42
SLC16A3	2.47	NYAP1	0.42
NDUFA4L2	2.31	MPIG6B	0.39
ECI1	2.28	GLP2R	0.39
IL11	2.23	CPM	0.38
PFKL	2.13	MTUS1	0.38
TGFBI	2.04	KCNK5	0.38
GAPDH	1.98	HMGCS1	0.37
TPI1	1.91	DDAH2	0.36
		PALM3	0.35
		TNFRSF11B	0.34
		GPR183	0.34
		LRRN1	0.31
		PIGZ	0.28
		EREG	0.27
		SLC6A12	0.12
		SLC4A11	0.07

**Table 6:** RNA sequencing: selected up- and down-regulated genes in bovine NP cells with FDR < 0.05 and p-value < 0.05 for 5 days of hypo-osmotic *versus* iso-osmotic treatment.

Top up-regulated targets		Top down-regulated targets	
Gene name	Fold change	Gene name	Fold change
TRIB3	6.01	IL4R	0.50
IL11	5.18	TLR4	0.47
TNFSF15	4.53	MMP2	0.46
TNFRSF21	4.30	SLC4A3	0.44
NGF	4.02	CD14	0.41
SLC7A11	3.94	SLC44A1	0.41
HAS2	3.79	SLC6A6	0.39
SLC7A5	3.74	MAP4K3	0.38
IL6	3.61	SLC37A2	0.37
ADAMTS9	3.55	ACAN	0.36
DDIT4	3.13	CD24	0.35
PTGS2	3.00	SLC13A4	0.34
COL1A1	2.92	SLC24A3	0.29
HSPA5	2.91	SLC46A3	0.27
SLC1A4	2.76	AQP1	0.26
COL1A2	2.74	ADAMTS12	0.22
VEGFC	2.66	CXCL5	0.21
SLC1A5	2.56	TNFRSF11B	0.21
SLC3A2	2.49	SLC39A8	0.20
VGF	2.15	SLC4A11	0.02

**Figure 5:**
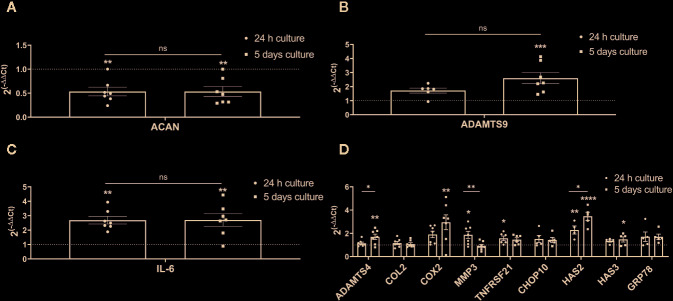
The mRNA expression of ECM and pro-inflammatory targets selected based on the RNA sequencing, novelty or relevance to the IVD degeneration in bovine NP cells: **(A)** aggrecan (ACAN), **(B)** ADAMTS9, **(C)** IL-6, and **(D)** ADAMTS4, COX2, HAS2, MMP3, COL2, TNFRSF21, CHOP10, HAS3, and GRP78. Graphs show 2^−ΔΔCt^ values normalized to the iso-osmotic control (mean ± SEM, bNP P2, n = 4–7). Asterisks indicate statistical significance (*p < 0.05, **p < 0.01, ***p < 0.001, ****p < 0.0001) between hypo-osmotic and iso-osmotic groups, bars compare the difference between 24 h and 5-day long groups (*ns*, no statistical difference defined at p > 0.05).

Aggrecan was significantly and steadily down-regulated in both 24 h and 5 days hypo-osmotic groups (24 h fold change: mean 0.53, min. 0.24, max. 1.00, p = 0.002; 5 day fold change: mean 0.53, min. 0.29, max. 1.00, p = 0.002; **Figure 5A**) as compared to iso-osmotic groups. ADAMTS9 was slightly up-regulated after 24 h of culture (24 h fold change: mean 1.72, min. 0.94, max. 2.24; **Figure 5B** left) and although the effect was statistically insignificant after 24 h (p = 0.15), its upregulation strongly increased over time and became significant (5 day fold change: mean 2.6, min. 1.45, max. 4.13, p = 0.001; **Figure 5B** right). Hypo-osmotic treatment consistently up-regulated IL-6 mRNA expression after 24 h (24 h fold change: mean 2.69, min. 1.89, max. 3.94, p = 0.002; **Figure 5C** left) and 5 days of culture (5 day fold change: mean 2.69, min. 0.90, max. 4.44, p = 0.002; **Figure 5C** right). However, due to the limited range of the ELISA kit, IL-6 protein release in the media could not be detected (data not showed). **Figure 5D** presents the gene expression of the remaining targets: ADAMTS4, COL2, COX2, MMP3, TNFRSF21, CHOP10, HAS2, HAS3, and GRP78. Although identified as potentially interesting targets on the small gene array data set, a more comprehensive analysis showed that hypo-osmotic treatment had little to no effect on the gene expression of ADAMTS4, COL2, TNFRSF21, CHOP10, HAS3, and GRP78. Interestingly, the gene expression of MMP3 decreased over time back to the iso-osmotic levels and the expression of COX2 and HAS2 was increased, especially at the later time point.

Finally, aggrecan, ADAMTS9 and IL-6 were chosen for further testing to check whether TRPV4 or TRPM7 channels may mediate their altered expression.

### Effects of TRPV4 and TRPM7 Activators on the Gene Expression of ACAN, ADAMTS9, IL-6, TRPV4, and TRPM7

In the following step, the effect of TRPV4 and TRPM7 compounds on the gene expression of aggrecan (ACAN), ADAMTS9, IL-6, TRPM7, and TRPV4 was tested. Bovine NP cells were cultured in the iso-osmotic, hypo-osmotic, or iso-osmotic media supplemented with a channel specific activator (TRPV4: 5 nM GSK1016790A or TRPM7: 25 µM Naltriben) over 24 h. The iso-osmotic activator-supplemented group was used to investigate 1) the influence of an activator on the gene expression of the tested channel, and 2) whether a TRP channel in question mediates up- or down-regulation of tested biomarker as observed during hypo-osmotic treatment alone.

**Figure 6:**
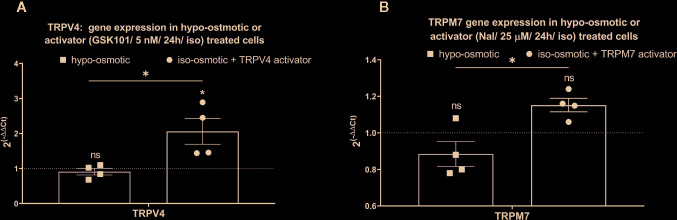
The mRNA expression of **(A)** TRPV4 in NP cells treated with 5 nM GSK1016790A, and **(B)** TRPM7 in NP cells treated with 25 µM Naltriben after 24 h. Graphs show 2^−ΔΔCt^ normalized to the iso-osmotic control (mean ± SEM, bNP P2, n = 4). Asterisks indicate statistical significance (*p < 0.05, no statistical difference defined at p > 0.05) between hypo-osmotic group, iso-osmotic group or iso-osmotic activator-supplemented group.

Addition of 5 nM TRPV4 activator to the iso-osmotic group up-regulated the expression of TRPV4 by around 2 fold (fold change: mean 2.06, min. 1.44, max. 2.89) in a statistically significant manner (p = 0.012) as compared to hypo-osmotic treatment alone (fold change: mean 0.91, min. 0.68, max. 1.1) and iso-osmotic control (p = 0.02) (**Figure 6A**). Iso-osmotic treatment with 25 µM TRPM7 activator slightly up-regulated TRPM7 expression (fold change: mean 1.15, min. 1.06, max. 1.24), with a statistical difference (p = 0.01) to the hypo-osmotic group (fold change: mean 0.86, min. 0.78, max. 1.08), but without a significant difference to the iso-osmotic group (p = 0.15) (**Figure 6B**).

**Figure 7:**
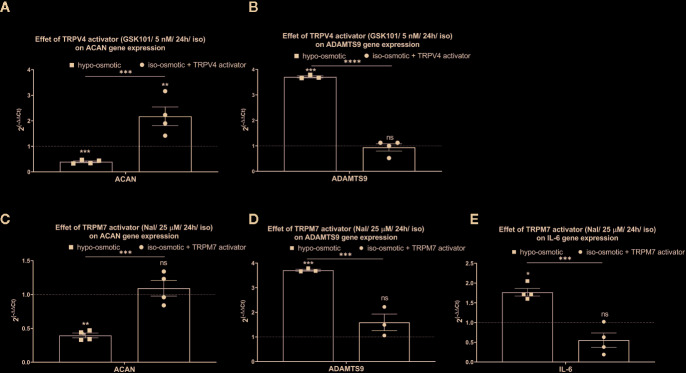
The mRNA expression of **(A)** aggrecan (ACAN) and **(B)** ADAMTS9 in NP cells after 24 h of treatment with TRPV4 activator (5 nM GSK1016790A); **(C)** aggrecan (ACAN), **(D)** ADAMTS9, and **(E)** IL-6 in NP cells after 24 h of treatment with TRPM7 activator (25 µM Naltriben). Graphs show 2^−ΔΔCt^ values normalized to the iso-osmotic control (mean ± SEM, bNP P2, n = 3–4). Asterisks indicate statistical significance (*p < 0.05, **p < 0.01, ***p < 0.001, ****p < 0.0001, *ns*, no statistical difference defined at p > 0.05) between hypo-osmotic group, iso-osmotic group or iso-osmotic activator-supplemented group.

While aggrecan was down-regulated and ADAMTS9 up-regulated under hypo-osmotic treatment (see also **Figure 5**), addition of GSK1016790A (TRPV4 activator) or Naltriben (TRPM7 activator) to iso-osmotic media did not yield a similar gene expression profile. GSK1016790A significantly up-regulated aggrecan expression (fold change: mean 2.18, min. 1.42, max. 3.16, p = 0.02; **Figure 7A** right), in contrast to hypo-osmotic treatment, which caused a significant down-regulation of aggrecan (fold change: mean 0.40, min. 0.33, max. 0.47, p = 0.0004; **Figure 7A** left), whereas Naltriben had no effect on aggrecan expression (fold change: mean 1.1, min. 0.84, max. 1.34, p = 0.8; **Figure 7C** right). Similarly, neither GSK1016790A nor Naltriben had an effect on ADAMTS9 expression (GSK101 group fold change: mean 0.94, min. 0.52, max. 1.12, p = 0.91, **Figure 7B** right and Naltriben group fold change: mean 1.60, min. 1.10, max. 2.22, p = 0.17; **Figure 7D** right) in contrary to the significant up-regulation observed in the hypo-osmotic group (fold change: mean 3.71, min. 3.70, max. 3.80, p = 0.0002; **Figures 7B** **, D** left). Hypo-osmotic treatment up-regulated IL-6 expression (fold change: mean 1.8, min. 1.60, max. 2.1, p = 0.01; **Figure 7E** left). While, TRPM7 activator gently down-regulated IL-6 expression (fold change: 0.56, min. 0.20, max. 1.00; **Figure 7E** right) as compared to the iso-osmotic group, but the effect was statistically insignificant (p = 0.1).

**Figure 8:**
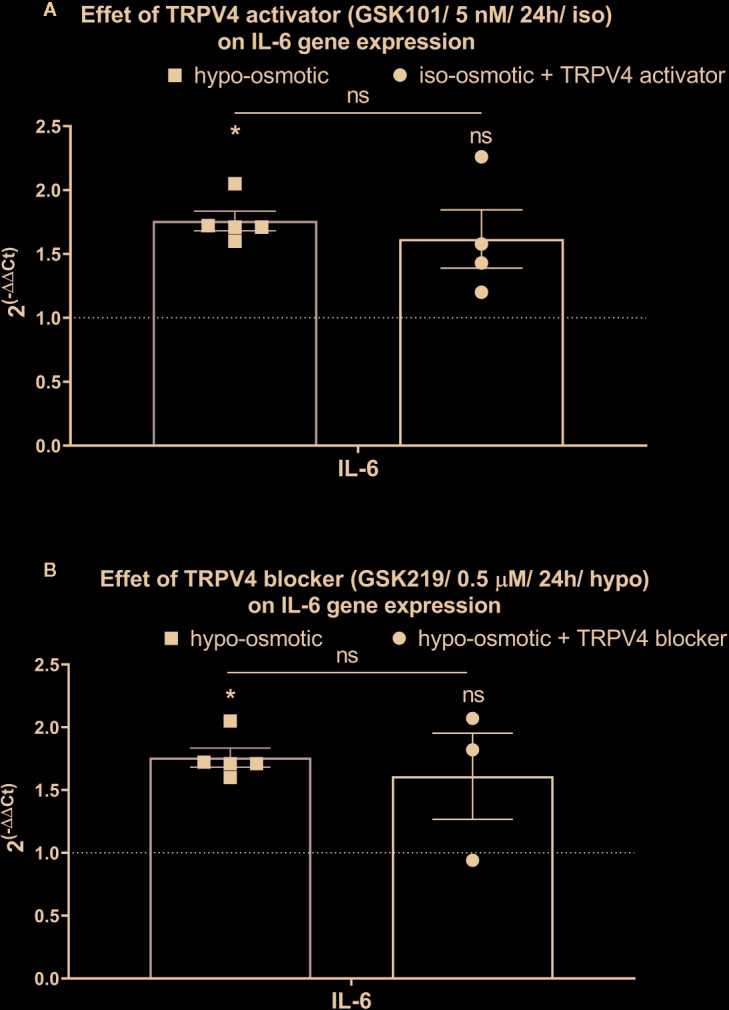
The mRNA expression of IL-6 in NP cells after 24 h of treatment with **(A)** TRPV4 activator (5 nM GSK1016790A) or **(B)** TRPV4 blocker (500 nM GSK2193874). Graphs show 2^−ΔΔCt^ values normalized to the iso-osmotic control (mean ± SEM, bNP P2, n = 3–4). Asterisks indicate statistical significance (*p < 0.05, *ns*, no statistical difference defined at p > 0.05) between hypo-osmotic group, iso-osmotic group, or iso-osmotic activator-supplemented group.

Interestingly, iso-osmotic GSK1016790A-supplementation seemed to up-regulate the expression of IL-6 (fold change: mean 1.62, min. 1.20, max. 2.30, p 0.11; **Figure 8A** right) in a manner similar to its expression measured in hypo-osmotic group (fold change: mean 1.8, min. 1.60, max. 2.1, p = 0.01; **Figure 8B** left). To further test if this phenomenon was mediated by TRPV4, TRPV4 blocker (500 nM GSK2193874) was added to hypo-osmotic media and cells were collected 24 h later. However, GSK2193874 did not prevent the upregulation of IL-6 in hypo-osmotic treatment (fold change: mean 1.61, min. 0.94, max. 2.1, p = 1; **Figure 8B** right), indicating that the hypo-osmotic induction of IL-6 is not (or only partially) mediated by TRPV4.

## Discussion

Discogenic back pain has a high prevalence in the western world and there is an increasing need for improved diagnostic and treatment strategies (Wieser et al., 2011; Geurts et al., 2018). TRP channels, which constitute a superfamily of ion channels and have been shown to be involved in tissue homeostasis and disease in many organs, might be a promising target for the treatment of low back pain (Krupkova et al., 2017; Sadowska et al., 2017; Sadowska et al., 2019). To our knowledge, this is the first study that presents a complete gene screening of TRP channels in bovine IVD cells, demonstrates the effects of osmotic stress on bovine NP cells over short (24 h) and long (5 days) culture times using RNA sequencing, as well as shows that TRPV4 or TRPM7 channels do not seem to mediate hypo-osmolarity induced changes in aggrecan, ADAMTS9, or IL-6 expression.

According to our results and similarly to the findings reported earlier on human IVD tissue (Sadowska et al., 2019), the three most highly expressed TRP channels were TRPC1, TRPV4, and TRPM7. TRPC1 belongs to the canonical (C) subfamily and is broadly expressed in mammalian tissues. TRPC1 is hypothesized to have a mechanosensitive function, can be stretch activated and it can interact with other TRP channel members, such as TRPV or TRPP (Du et al., 2014). Decreased TRPC1 protein expression was linked to a greater increase in liver cells volume (Chen and Barritt, 2003), indicating that TRPC1 may be a potential candidate for the investigation of osmosensing and volume regulation. We could demonstrate that TRPC1 was consistently well expressed in bovine NP and AF cells, and its expression was unaffected by passaging, which is consistent with earlier results on human non-degenerated NP and AF tissue (Sadowska et al., 2019) as well as on human articular chondrocytes (Gavenis et al., 2009). However, due to the lack of TRPC1 channel specific activators and blockers, this channel was excluded from further research in the present study.

Osmosensing properties of the TRPV4 channel were investigated in numerous studies including tissues such as kidney, DRG neurons, epithelium, cartilage as well as IVD (Alessandri-Haber et al., 2003; Liedtke and Friedman, 2003; Mizuno et al., 2003; Arniges et al., 2004; Andrade et al., 2005; Cohen, 2005; Liedtke, 2005; Plant and Strotmann, 2007; Walter et al., 2016). Although limited data on TRPV4 in the IVD exists, it is believed to be the key mechano- and osmosensor protein in cartilage (Phan et al., 2009; Clark et al., 2010; Jablonski et al., 2014; O’Conor et al., 2014; Servin-Vences et al., 2017). It was previously demonstrated that in chondrocytes TRPV4 may interact with integrin α1β1 in response to hypo-osmotic stress (wildtype and integrin α1-null mice) (Jablonski et al., 2014), participate in cell volume regulation (porcine articular chondrocytes) (Phan et al., 2009), enhance ECM accumulation (porcine articular chondrocytes) (O’Conor et al., 2014) and its expression may be mediated by ERK_1/2_ signaling (equine articular chondrocytes) (Hdud et al., 2014). However, NP cells, despite being morphologically similar to articular chondrocytes, have distinctively different ECM composition and biomechanical properties, which should be considered when comparing both cell types (Aota et al., 2006; Lee et al., 2007; Cui et al., 2010). In our study, TRPV4 was overall the most highly expressed TRP channel in freshly isolated NP and AF cells, but its expression significantly decreased with passaging. A previous study reported comparable TRPV4 gene expression levels between degenerated and non-degenerated human IVD tissue, independent of tissue or patient specific characteristics, suggesting that TRPV4 could play a fundamental role in the IVD homeostasis (Sadowska et al., 2019). A previous study reported increased TRPV4 protein expression in bovine NP cells after hypo-osmotic stimulation (Walter et al., 2016), however we were not able to reproduce these results. Another study demonstrated that TRPV4 gene expression seemed to be down-regulated, but without reaching statistical significance, under hypo-osmotic (130 mOsm/l) burst (1.5 h/day) and cyclic (10 min on and off for 1.5 h/day) conditions as compared to static loading (24 h/day) in mice NP organ culture model (Palacio-Mancheno et al., 2017). In line with the latter (Palacio-Mancheno et al., 2017), but in contrary to the earlier study (Walter et al., 2016), we showed that TRPV4 gene expression did not significantly differ between 24 h and 5 days of hypo-osmotic treatment and was significantly down-regulated as compared to iso-osmotic treatment. However, we have also noticed donor-to-donor variations in TRPV4 (and TRPM7) gene expression. The contradictory findings between this and the past study (Walter et al., 2016) may be partially due to different detection techniques used, namely western blot *versus* RNA sequencing, qPCR, and ICC (our study). In fact, we also intended to test TRPV4 protein expression using western blot to enhance comparability between studies, but found that antibodies available at the time of the analysis were, in our hands, non-specific for bovine cells as discerned in preliminary tests (data not shown).

Although TRPV4 expression is certainly of interest, changes in channel activity are ultimately of higher relevance in the context of sensing environmental changes and calcium signaling. Therefore, in the next step we employed GSK1016790A (Thorneloe et al., 2008; Jin et al., 2011) and GSK2193874 (Thorneloe et al., 2012; Cheung et al., 2017), which are currently amongst the most selective TRPV4 channel activators and blockers, respectively. GSK1016790A was shown to induce Ca^2+^ influx through TRPV4 channel with estimated EC_50_ values of 2.1 nM (HEK293-hTRPV4 cells), 11 nM (guinea-pig urothelial cells), and 18 nM (HEK293- mTRPV4 cells) (Thorneloe et al., 2008; Xu et al., 2009), while GSK2193874 inhibits Ca^2+^ influx mediated by TRPV4 channels with IC_50_ values of 40–50 nM (HEK293-hTRPV4 cells) and 2 nM (HEK293-rTRPV4 cells) (Thorneloe et al., 2012; Cheung et al., 2017). In recent publications, it was demonstrated that stimulation with GSK1016790A causes a down-regulation of TRPV4 channels from the plasma membrane within 20–30 min post-treatment (10 nM in HeLa-TRPV4 cells (Jin et al., 2011) and 100 nM in HUVECs (Baratchi et al., 2019)) and translocation of the TRPV4 channel to the recycling endosomes (Baratchi et al., 2019). We first tested whether there are any differences in TRPV4 channel activity properties between hypo- and iso-osmotic conditions and secondly whether the TRPV4 channel mediates gene expression changes of targets differently regulated by hypo-osmotic stress. When GSK1016790A was applied to both hypo- and iso-osmotic treated cells, a higher calcium flux was measured in the hypo-osmotic group, indicating that TRPV4 activity in bovine NP cells may be higher at reduced osmolarity. The mechanism of TRPV4 activation is still being discussed and several modes of action have been proposed. TRPV4 can be spontaneously activated at physiological osmolarity levels and can respond to changes in the local environment (increase its activity at reduced osmolarity and *vice versa*). In this context, TRPV4 activation can be triggered by cell volume changes, but presumably not directly due to stretch, but rather due to the activation of phospholipase A2 (PLA2) (Plant and Strotmann, 2007; Pedersen and Nilius, 2007; Servin-Vences et al., 2017). However, it is worth mentioning that differentiating between mechano- and osmo-function can be challenging, since osmotic cell swelling is inherently associated with membrane stretch. Interestingly, it was shown that in salivary glands, the activity of TRPV4 may be dependent on AQP5 (Liu et al., 2006), which therefore points towards a possibly complex osmosensing network.

The second most abundantly expressed TRP channel in bovine NP cells was TRPM7. TRPM7 belongs to the TRP melastatin (M) subfamily that is most recognized for its role in thermo-sensation, but also seems to be implicated in cell survival/death, cytokine release, and response to oxidative stress (McNulty and Fonfria, 2005; Massullo et al., 2006). In certain tissue types that are known to experience daily osmotic changes, such as kidney, TRPM7 was identified as a mechano- and osmosensor that is involved in the regulation of cell volume changes (Numata et al., 2007; Kim et al., 2017). TRPM7 was so far only sparsely investigated in the IVD: TRPM7 was previously detected in non-degenerated and degenerated IVD tissue, where its expression tended to increase with the IVD degeneration grade (Sadowska et al., 2019). In this study, we could demonstrate that TRPM7 was well expressed in both, bovine NP and AF cells, showed less variation than TRPV4 in its expression between passaging and its expression was unaffected by the hypo-osmotic treatment. This is partially in line with an earlier study on human articular chondrocytes, where TRPM7 was highly expressed in non-passaged cells, but contrary to our study, its expression greatly diminished with passaging (Gavenis et al., 2009). Naltriben, with an EC_50_ 20 µM (HEK 293-mTRPM7 (Hofmann et al., 2014)), 24 µM (rat ventricular myocytes (Tashiro et al., 2019)), and 45 µM (ameloblast cell line LS8 cells (Souza Bomfim et al., 2020)), and NS8593, with an IC_50_ 1.6 µM (HEK 293 cells (Chubanov et al., 2012)) and 2 µM (rat ventricular myocytes (Tashiro et al., 2014)), are compounds that respectively activate and block TRPM7 channel. Naltriben was initially characterized as an antagonist of δ-opioid receptors (June et al., 1999) and may compete with the inhibitory effect of NS8598, but does not stimulate other TRP channels including TRPM2, TRPM3, TRPM8, and TRPV1 (Hofmann et al., 2014). NS8593 is a potent TRPM7 inhibitor that exhibits complete and reversible block of TRPM7 currents, but may also target small conductance potassium channels, which are not related to TRPM7 channel (Jenkins et al., 2011; Chubanov et al., 2012). Inhibitory action of NS8593 on native TRPM7-like currents was demonstrated among others in HEK 293 cells, freshly isolated smooth muscle cells, ventricular myocytes, as well as in primary podocytes (Chubanov et al., 2012). It was shown that 50 µM Naltriben can effectively activate TRPM7 channel (without measurable off-target responses) and this response can be blocked with 20 µM NS8593 in HEK 293 cells overexpressing TRPM7 (Hofmann et al., 2014). Moreover, 100 µM Naltriben activated and 100 µM NS8593 blocked Ca^2+^ fluctuations in mice chondrocytes from femoral cartilage plate (Qian et al., 2019). In contrast, in the presented study we have used Naltriben concentrations ranging from 25 to 200 µM depending on the duration of the treatment and assay type. We have furthermore observed that an application of 50 µM Naltriben was not able to induce Ca^2+^ flux in primary bovine NP cells, while 100 µM had only minor effect as compared to the baseline (**Supplementary Figure 2**). Furthermore, we have not observed differences in Ca^2+^ flux between the hypo- and iso-osmotic conditions, but the scientific evidence for TRPM7 sensitivity to osmotic gradient is inconclusive and suggests that observed differences may depend on the cell type. For example, it was shown that TRPM7 is involved in regulatory volume decrease (RVD) after osmotic cell swelling and TRPM7 silencing reduced the rate of RVD in human HeLa and human embryonic kidney HEK293T cells (Numata et al., 2007), hence indicating TRPM7’s sensitivity to osmotic changes. Yet, TRPM7 current was unaffected by hypotonic solution or cell swelling in rat brain microglia (Jiang et al., 2003). Overall, our data indicate that TRPM7 gene/protein expression as well as activity is independent from hypo-osmotic stress in bovine NP cells.

Under hypo-osmotic stimulation, NP cells exhibited a significant increase in mRNA levels of pro-inflammatory and catabolic factors, such as IL-6 and IL-11, Small-Inducible Cytokine A5 (CCL5), nerve growth factor (NGF), ADAMTS4 and ADAMTS9, and factors inducing cell apoptosis *via* nuclear factor kappa-B (NF-κB), e.g. Tumor Necrosis Factor Receptor Superfamily Member 21 (TNFRSF21) or Neuronal Cell Death Inducible Putative Kinase (TRIB3). In contrast, a down-regulation was observed for several membrane proteins involved in transporting soluble molecules (e.g. members of solute carrier family [SCL]) and water channels (e.g. aquaporin [AQP] especially AQP1), as well as, aggrecan (ACAN). IL-6, ADAMTS9, and aggrecan were selected to test whether their altered gene expression observed under hypo-osmotic stimulation may be mediated by the TRPV4 or TRPM7 channel. IL-6 is a widely investigated cytokine in the field of painful disc degeneration. Increased IL-6 expression was observed in herniated and degenerated IVDs (Lee et al., 2009). Moreover, IL-6 can be secreted by IVD cells and induce TNF-α expression, which is associated with neuropathic pain (Risbud and Shapiro, 2014; Weber et al., 2016). Hence, targeting IL-6 may have beneficial effects for pain treatment. Aggrecan is the primary proteoglycan of NP cells, is important for the normal osmotic function of the IVD as it provides the ability to bind water, and is therefore contributing to the tissue’s hydration, integrity, and its biomechanical function (e.g. ability to withstand load). In contrast, ADAMTS9 is an aggrecanase, which together with other members of the ADAMTS family, mediates aggrecan turnover and degradation in the IVD. It was established that ADAMTS9 is expressed in non-degenerated and degenerated IVD tissue, with an increased protein expression in the latter (Pockert et al., 2009). Moreover, ADAMTS9 was shown to be induced by IL-1β and TNF-α in human chondrocytes (Demircan et al., 2005). We have demonstrated that hypo-osmotic treatment alone increases the expression of IL-6 and ADAMTS9 and decreases the expression of aggrecan in healthy bovine NP cells.

A past study has demonstrated that activation of TRPV4 with GSK1016790A may induce ECM and immune system regulatory gene expression changes (e.g. down-regulation of ADAMTS5 and NOS2) similarly to dynamic loading alone and increases ECM accumulation akin to a long-term (≥2 weeks) osmotic loading in porcine articular chondrocytes (O’Conor et al., 2014). However, in the same study TRPV4 inhibition with GSK205 alone (that is without the dynamic loading) had no effect on the gene expression of tested targets (O’Conor et al., 2014). Yet in another study, TRPV4 inhibition with GSK2193874 reduced cytokine production (TNFα, IL-1α, and IL-6) in septic mice (Dalsgaard et al., 2016). Here we have shown that activation of TRPV4 with GSK1016790A in the iso-osmotic condition up-regulated aggrecan (but inversely to the hypo-osmotic treatment), had no effect on ADAMTS9 expression and induced IL-6 in a manner similar to hypo-osmotic treatment alone. However, inhibition of TRPV4 activity in bovine NP cells was not able to reverse the hypo-osmotically-induced IL-6 expression, although past studies on bovine NP cells hypothesized that IL-6 is a downstream target of TRPV4 (Walter et al., 2016). Therefore, we speculate that the hypo-osmotically-induced gene changes of IL-6, aggrecan, and ADAMTS9 are not mediated by TRPV4 in the IVD. Future studies should examine the interplay between inflammation and TRPV4 and TRPM7 sensitization and activity in the IVD. The observed IL-6 upregulation may be augmented in part by prostanoid PGE2 activation of PAR-2 signaling (Poole et al., 2013), which was shown to cause TRPV4 sensitization in mice (Alessandri-Haber et al., 2005) and rats (Alessandri-Haber et al., 2004) in studies on inflammatory and neuropathic pain as well as mechanical hyperalgesia (Grant et al., 2007). Moreover, our data suggests that TRPM7 activation does not influence aggrecan, ADAMTS9 or IL-6 expression alike to hypo-osmotic treatment. Interestingly, it was shown that IL-6 might inhibit TRPM7 currents *via* JAK2-STAT3 signaling pathways in rat cortical neurons (Liu et al., 2016). Furthermore, it was indicated that TRPM7 inhibition (with a non-specific channel inhibitor 2-APB) decreased IL-6 release in allergen‐sensitized rat BMMC (Huang et al., 2014). Thus, there is a possible relationship between IL-6 and TRPM7, whereas it might not be associated with osmotic loading alone and may be tissue/cell type dependent. Future studies will aim to establish whether the interplay of TRP channels with other membrane proteins may contribute to the altered expression of these and other targets under hypo-osmotic stimulation. A limitation of the current experimental design was the use of bovine IVD tissue instead of healthy human IVD tissue, which may in the future be beneficial for translating current findings. Moreover, in contrary to bovine tails, human spine experiences day-to-day mechanical loading from body weight and daily activities, which not only can act as a stimuli for IVD cells and TRP channels, but also lead to daily osmotic shifts (e.g. increased osmotic pressure during daily life activities and reduced osmotic pressure during rest periods) (Schmidt et al., 2010; Chan et al., 2011; Sadowska et al., 2018). Hence, dynamic mechanical loading as well as diurnal osmotic loading could be incorporated in the future studies. Additionally, a lack of channel specific activators and blockers (e.g. for TRPC1) and possible off-target effects for Naltriben and NS8593 limited the scope of this project.

## Conclusion

In summary, we demonstrated that several TRP channels are expressed in bovine IVD cells. Hypo-osmotic treatment led to a higher calcium flux through TRPV4. We have presented genome-wide expression changes caused by reduced osmolarity in bovine NP cells and have shown through pharmacological activation and/or inhibition of TRPV4 and TRPM7 that these channels likely do not mediate hypo-osmotically-induced gene expression changes of aggrecan, ADAMTS9, and IL-6 in bovine NP cells.

## Data Availability Statement

The datasets presented in this study can be found in online repositories. The names of the repository and accession number can be found here: https://www.ebi.ac.uk/ena, PRJEB37486.

## Ethics Statement

Ethics approval was not required for experiments using animal tissue according to national legislation and institutional guidelines.

## Author Contributions

AS designed and executed cell experiments, analyzed and interpreted data, prepared the graphs, and drafted the manuscript. BA performed the calcium imaging experiments. WH conducted statistical analysis on the gene array and calcium data and proof-read the manuscript. SF participated in the study design and critically reviewed the manuscript. KW-K contributed to the conception of the study, provided expertise, secured the funding, and helped drafting the manuscript.

## Funding

The study was financially supported by the Swiss Neuro Foundation (Bern/Switzerland), the Swiss National Science Foundation (SNF PP00P2_163678), as well as the Spine Society of Europe (Eurospine 2016_4).

## Conflict of Interest

The authors declare that the research was conducted in the absence of any commercial or financial relationships that could be construed as a potential conflict of interest.

## Supplementary Material

Supplementary Table 1 Summary of GSEA results with FDR <0.05 and p-value <0.05 for the 24 h treatment group of hypo-osmotic *versus* iso-osmotic stimulation in bovine NP cells.

Supplementary Table 2 Summary of GSEA results with FDR <0.05 and p-value <0.05 for the 5 days long treatment group of hypo-osmotic *versus* iso-osmotic stimulation in bovine NP cells.

## References

[B1] Alessandri-Haber N. Yeh J. J. Boyd A. E. Parada C. A. Chen X. Reichling D. B. (2003). Hypotonicity induces TRPV4-mediated nociception in rat. Neuron 39, 497–511. 10.1016/S0896-6273(03)00462-8 12895423

[B2] Alessandri-Haber N. Dina O. A. Yeh J. J. Parada C. A. Reichling D. B. Levine J. D. (2004). Transient receptor potential vanilloid 4 is essential in chemotherapy-induced neuropathic pain in the rat. J. Neurosci. 24, 4444–4452. 10.1523/JNEUROSCI.0242-04.2004 15128858 PMC6729449

[B3] Alessandri-Haber N. Joseph E. Dina O. A. Liedtke W. Levine J. D. (2005). TRPV4 mediates pain-related behavior induced by mild hypertonic stimuli in the presence of inflammatory mediator. Pain 118, 70–79. 10.1016/j.pain.2005.07.016 16213085

[B4] Andrade Y. N. Fernandes J. Vazquez E. Fernandez-Fernandez J. M. Arniges M. Sanchez T. M. (2005). TRPV4 channel is involved in the coupling of fluid viscosity changes to epithelial ciliary activity. J. Cell Biol. 168, 869–874. 10.1083/jcb.200409070 15753126 PMC2171792

[B5] Aota Y. An H. S. Imai Y. Thonar E. J. Muehleman C. Masuda K. (2006). Comparison of cellular response in bovine intervertebral disc cells and articular chondrocytes: effects .of lipopolysaccharide on proteoglycan metabolism. Cell Tissue Res. 326 , 787–93. 10.1007/s00441-006-0225-1 16788835

[B6] Arniges M. Vazquez E. Fernandez-Fernandez J. M. Valverde M. A. (2004). Swelling-activated Ca2+ entry via TRPV4 channel is defective in cystic fibrosis airway epithelia. J. Biol. Chem. 279, 54062–54068. 10.1074/jbc.M409708200 15489228

[B7] Baratchi S. Keov P. Darby W. G. Lai A. Khoshmanesh K. Thurgood P. (2019). The TRPV4 Agonist GSK1016790A Regulates the Membrane Expression of TRPV4 Channels. Front. Pharmacol. 10, 6. 10.3389/fphar.2019.00006 30728775 PMC6351496

[B8] Bolger A. M. Lohse M. Usadel B. (2014). Trimmomatic: a flexible trimmer for Illumina sequence data. Bioinformatics 30, 2114–2120. 10.1093/bioinformatics/btu170 24695404 PMC4103590

[B9] Bray N. L. Pimentel H. Melsted P. Pachter L. (2016). Near-optimal probabilistic RNA-seq quantification. Nat. Biotechnol. 34, 525–527. 10.1038/nbt.3519 27043002

[B10] Cassinelli E. H. Hall R. A. Kang J. D. (2001). Biochemistry of intervertebral disc degeneration and the potential for gene therapy applications. Spine J. 1, 205–214. 10.1016/S1529-9430(01)00021-3 14588349

[B11] Chan S. C. Ferguson S. J. Gantenbein-Ritter B. (2011). The effects of dynamic loading on the intervertebral disc. Eur. Spine J. 20, 1796–1812. 10.1007/s00586-011-1827-1 21541667 PMC3207351

[B12] Chen J. Barritt G. J. (2003). Evidence that TRPC1 (transient receptor potential canonical 1) forms a Ca(2+)-permeable channel linked to the regulation of cell volume in liver cells obtained using small interfering RNA targeted against TRPC1. Biochem. J. 373, 327–336. 10.1042/bj20021904 12720547 PMC1223516

[B13] Cheung M. Bao W. Behm D. J. Brooks C. A. Bury M. J. Dowdell S. E. (2017). Discovery of GSK2193874: An Orally Active, Potent, and Selective Blocker of Transient Receptor Potential Vanilloid 4. ACS Med. Chem. Lett. 8, 549–554. 10.1021/acsmedchemlett.7b00094 28523109 PMC5430398

[B14] Chubanov V. Mederos y Schnitzler M. Meissner M. Schafer S. Abstiens K. Hofmann T. (2012). Natural and synthetic modulators of SK (K(ca)2) potassium channels inhibit magnesium-dependent activity of the kinase-coupled cation channel TRPM7. Br. J. Pharmacol. 166, 1357–1376. 10.1111/j.1476-5381.2012.01855.x 22242975 PMC3417452

[B15] Clark A. L. Votta B. J. Kumar S. Liedtke W. Guilak F. (2010). Chondroprotective role of the osmotically sensitive ion channel transient receptor potential vanilloid 4: age- and sex-dependent progression of osteoarthritis in Trpv4-deficient mice. Arthritis Rheum. 62, 2973–2983. 10.1002/art.27624 20583100 PMC3027356

[B16] Cohen D. M. (2005). TRPV4 and the mammalian kidney. Pflugers Arch. 451, 168–175. 10.1007/s00424-005-1456-9 15988590

[B17] Cui Y. Yu J. Urban J. P. Young D. A. (2010). Differential gene expression profiling of metalloproteinases and their inhibitors: a comparison between bovine intervertebral disc nucleus pulposus cells and articular chondrocytes. Spine (Phila Pa 1976) 35 , 1101–118. 10.1097/BRS.0b013e3181c0c727 20473119

[B18] Dalsgaard T. Sonkusare S. K. Teuscher C. Poynter M. E. Nelson M. T. (2016). Pharmacological inhibitors of TRPV4 channels reduce cytokine production, restore endothelial function and increase survival in septic mice. Sci. Rep. 6, 33841. 10.1038/srep33841 27653046 PMC5031985

[B19] Demers C. N. Antoniou J. Mwale F. (2004). Value and limitations of using the bovine tail as a model for the human lumbar spine. Spine (Phila Pa 1976) 29 , 2793–279. 10.1097/01.brs.0000147744.74215.b0 15599281

[B20] Demircan K. Hirohata S. Nishida K. Hatipoglu O. F. Oohashi T. Yonezawa T. (2005). ADAMTS-9 is synergistically induced by interleukin-1beta and tumor necrosis factor alpha in OUMS-27 chondrosarcoma cells and in human chondrocytes. Arthritis Rheum. 52, 1451–1460. 10.1002/art.21010 15880812

[B21] Dobin A. Davis C. A. Schlesinger F. Drenkow J. Zaleski C. Jha S. (2013). STAR: ultrafast universal RNA-seq aligner. Bioinformatics 29, 15–21. 10.1093/bioinformatics/bts635 23104886 PMC3530905

[B22] Du J. Ma X. Shen B. Huang Y. Birnbaumer L. Yao X. (2014). TRPV4, TRPC1, and TRPP2 assemble to form a flow-sensitive heteromeric channel. FASEB J. 28, 4677–4685. 10.1096/fj.14-251652 25114176 PMC4200325

[B23] Gavenis K. Schumacher C. Schneider U. Eisfeld J. Mollenhauer J. Schmidt-Rohlfing B. (2009). Expression of ion channels of the TRP family in articular chondrocytes from osteoarthritic patients: changes between native and in vitro propagated chondrocytes. Mol. Cell Biochem. 321, 135–134. 10.1007/s11010-008-9927-x 18836817

[B24] Geurts J. W. Willems P. C. Kallewaard J. W. van Kleef M. Dirksen C. (2018). The Impact of Chronic Discogenic Low Back Pain: Costs and Patients’ Burden. Pain Res. Manag. 2018, 4696180. 10.1155/2018/4696180 30364097 PMC6188764

[B25] Gomis A. Soriano S. Belmonte C. Viana F. (2008). Hypoosmotic- and pressure-induced membrane stretch activate TRPC5 channels. J. Physiol. 586, 5633–5649. 10.1113/jphysiol.2008.161257 18832422 PMC2655392

[B26] Grant A. D. Cottrell G. S. Amadesi S. Trevisani M. Nicoletti P. Materazzi S. (2007). Protease-activated receptor 2 sensitizes the transient receptor potential vanilloid 4 ion channel to cause mechanical hyperalgesia in mice. J. Physiol. 578, 715–733. 10.1113/jphysiol.2006.121111 17124270 PMC2151332

[B27] Grimm C. Kraft R. Sauerbruch S. Schultz G. Harteneck C. (2003). Molecular and functional characterization of the melastatin-related cation channel TRPM3. J. Biol. Chem. 278, 21493–21501. 10.1074/jbc.M300945200 12672799

[B28] Harteneck C. Reiter B. (2007). TRP channels activated by extracellular hypo-osmoticity in epithelia. Biochem. Soc. Trans. 35, 91–95. 10.1042/BST0350091 17233610

[B29] Hdud I. M. Mobasheri A. Loughna P. T. (2014). Effect of osmotic stress on the expression of TRPV4 and BKCa channels and possible interaction with ERK1/2 and p38 in cultured equine chondrocytes. Am. J. Physiol. Cell Physiol. 306, C1050–C1057. 10.1152/ajpcell.00287.2013 24671100

[B30] Hofmann T. Schafer S. Linseisen M. Sytik L. Gudermann T. Chubanov V. (2014). Activation of TRPM7 channels by small molecules under physiological conditions. Pflugers Arch. 466, 2177–2189. 10.1007/s00424-014-1488-0 24633576

[B31] Huang L. Ng N. M. Chen M. Lin X. Tang T. Cheng H. (2014). Inhibition of TRPM7 channels reduces degranulation and release of cytokines in rat bone marrow-derived mast cells. Int. J. Mol. Sci. 15, 11817–11831. 10.3390/ijms150711817 24995695 PMC4139816

[B32] Jablonski C. L. Ferguson S. Pozzi A. Clark A. L. (2014). Integrin alpha1beta1 participates in chondrocyte transduction of osmotic stress. Biochem. Biophys. Res. Commun. 445, 184–190. 10.1016/j.bbrc.2014.01.157 24495803 PMC4022045

[B33] Jenkins D. P. Strobaek D. Hougaard C. Jensen M. L. Hummel R. Sorensen U. S. (2011). Negative gating modulation by (R)-N-(benzimidazol-2-yl)-1,2,3,4-tetrahydro-1-naphthylamine (NS8593) depends on residues in the inner pore vestibule: pharmacological evidence of deep-pore gating of K(Ca)2 channels. Mol. Pharmacol. 79, 899–909. 10.1124/mol.110.069807 21363929 PMC3102549

[B34] Jiang X. Newell E. W. Schlichter L. C. (2003). Regulation of a TRPM7-like current in rat brain microglia. J. Biol. Chem. 278, 42867–42876. 10.1074/jbc.M304487200 12904301

[B35] Jin M. Wu Z. Chen L. Jaimes J. Collins D. Walters E. T. (2011). Determinants of TRPV4 activity following selective activation by small molecule agonist GSK1016790A. PloS One 6, e16713. 10.1371/journal.pone.0016713 21339821 PMC3038856

[B36] June H. L. McCane S. R. Zink R. W. Portoghese P. S. Li T. K. Froehlich J. C. (1999). The delta 2-opioid receptor antagonist naltriben reduces motivated responding for ethanol. Psychopharmacol. (Berl.) 147 , 81–9. 10.1007/s002130051145 10591872

[B37] Kim J. M. Choi S. Park K. (2017). TRPM7 Is Involved in Volume Regulation in Salivary Glands. J. Dent. Res. 96, 1044–1050. 10.1177/0022034517708766 28499095

[B38] Koc Z. Ozcakir S. Sivrioglu K. Gurbet A. Kucukoglu S. (2009). Effectiveness of physical therapy and epidural steroid injections in lumbar spinal stenosis. Spine (Phila Pa 1976) 34 , 985–99. 10.1097/BRS.0b013e31819c0a6b 19404172

[B39] Krupkova O. Zvick J. Wuertz-Kozak K. (2017). The role of transient receptor potential channels in joint diseases. Eur. Cell Mater. 34, 180–201. 10.22203/eCM.v034a12 28994450

[B40] Le Maitre C. L. Freemont A. J. Hoyland J. A. (2004). Localization of degradative enzymes and their inhibitors in the degenerate human intervertebral disc. J. Pathol. 204, 47–54. 10.1002/path.1608 15307137

[B41] Lee C. R. Sakai D. Nakai T. Toyama K. Mochida J. Alini M. (2007). A phenotypic comparison of intervertebral disc and articular cartilage cells in the rat. Eur. Spine J. 16, 2174–2185. 10.1007/s00586-007-0475-y 17786487 PMC2140128

[B42] Lee S. Moon C. S. Sul D. Lee J. Bae M. Hong Y. (2009). Comparison of growth factor and cytokine expression in patients with degenerated disc disease and herniated nucleus pulposus. Clin. Biochem. 42, 1504–1511. 10.1016/j.clinbiochem.2009.06.017 19563795

[B43] Liedtke W. Friedman J. M. (2003). Abnormal osmotic regulation in trpv4-/- mice. Proc. Natl. Acad. Sci. U.S.A. 100 , 13698–1703. 10.1073/pnas.1735416100 PMC263876 14581612

[B44] Liedtke W. (2005). TRPV4 as osmosensor: a transgenic approach. Pflugers Arch. 451, 176–180. 10.1007/s00424-005-1449-8 15952033

[B45] Liu X. Bandyopadhyay B. C. Nakamoto T. Singh B. Liedtke W. Melvin J. E. (2006). A role for AQP5 in activation of TRPV4 by hypotonicity: concerted involvement of AQP5 and TRPV4 in regulation of cell volume recovery. J. Biol. Chem. 281, 15485–15495. 10.1074/jbc.M600549200 16571723

[B46] Liu A. Zhao F. Wang J. Zhao Y. Luo Z. Gao Y. (2016). Regulation of TRPM7 Function by IL-6 through the JAK2-STAT3 Signaling Pathway. PloS One 11, e0152120. 10.1371/journal.pone.0152120 27010689 PMC4806911

[B47] Massullo P. Sumoza-Toledo A. Bhagat H. Partida-Sánchez S. (2006). TRPM channels, calcium and redox sensors during innate immune responses, Seminars in cell & developmental biology. Elsevier, 17 (6), 654–666. 10.1016/j.semcdb.2006.11.006 17178241

[B48] McNulty S. Fonfria E. (2005). The role of TRPM channels in cell death. Pflugers Arch. 451, 235–242. 10.1007/s00424-005-1440-4 16025303

[B49] Mizuno A. Matsumoto N. Imai M. Suzuki M. (2003). Impaired osmotic sensation in mice lacking TRPV4. Am. J. Physiol. Cell Physiol. 285, C96–101. 10.1152/ajpcell.00559.2002 12777254

[B50] Molinos M. Almeida C. R. Caldeira J. Cunha C. Goncalves R. M. Barbosa M. A. (2015). Inflammation in intervertebral disc degeneration and regeneration. J. R. Soc. Interface 12, 20141191. 10.1098/rsif.2015.0429 25673296 PMC4345483

[B51] Numata T. Shimizu T. Okada Y. (2007). TRPM7 is a stretch- and swelling-activated cation channel involved in volume regulation in human epithelial cells. Am. J. Physiol. Cell Physiol. 292, C460–C467. 10.1152/ajpcell.00367.2006 16943238

[B52] O’Conor C. J. Leddy H. A. Benefield H. C. Liedtke W. B. Guilak F. (2014). TRPV4-mediated mechanotransduction regulates the metabolic response of chondrocytes to dynamic loading. Proc. Natl. Acad. Sci. U.S.A. 111, 1316–1321. 10.1073/pnas.1319569111 24474754 PMC3910592

[B53] Palacio-Mancheno P. E. Evashwick-Rogler T. W. Laudier D. M. Purmessur D. Iatridis J. C. (2017). Hyperosmolarity induces notochordal cell differentiation with aquaporin3 upregulation and reduced N-cadherin expression. J. Orthop. Res. 36 (2), 788–798. 10.1002/jor.23715 28853179 PMC5832547

[B54] Pedersen S. F. Nilius B. (2007). Transient receptor potential channels in mechanosensing and cell volume regulation. Methods Enzymol. 428, 183–207. 10.1016/S0076-6879(07)28010-3 17875418

[B55] Phan M. N. Leddy H. A. Votta B. J. Kumar S. Levy D. S. Lipshutz D. B. (2009). Functional characterization of TRPV4 as an osmotically sensitive ion channel in porcine articular chondrocytes. Arthritis Rheum. 60, 3028–3037. 10.1002/art.24799 19790068 PMC2846816

[B56] Plant T. D. Strotmann R. (2007). “TRPV4,” in Transient Receptor Potential (TRP) Channels. Eds. Flockerzi V. Nilius B. (Berlin, Heidelberg: Springer Berlin Heidelberg), 189–205.

[B57] Pockert A. J. Richardson S. M. Le Maitre C. L. Lyon M. Deakin J. A. Buttle D. J. (2009). Modified expression of the ADAMTS enzymes and tissue inhibitor of metalloproteinases 3 during human intervertebral disc degeneration. Arthritis Rheum. 60, 482–491. 10.1002/art.24291 19180493

[B58] Poole D. P. Amadesi S. Veldhuis N. A. Abogadie F. C. Lieu T. Darby W. (2013). Protease-activated receptor 2 (PAR2) protein and transient receptor potential vanilloid 4 (TRPV4) protein coupling is required for sustained inflammatory signaling. J. Biol. Chem. 288, 5790–5802. 10.1074/jbc.M112.438184 23288842 PMC3581372

[B59] Qian N. Ichimura A. Takei D. Sakaguchi R. Kitani A. Nagaoka R. (2019). TRPM7 channels mediate spontaneous Ca(2+) fluctuations in growth plate chondrocytes that promote bone development. Sci. Signal 12, eaaw4847. 10.1126/scisignal.aaw4847 30967513

[B60] Risbud M. V. Shapiro I. M. (2014). Role of cytokines in intervertebral disc degeneration: pain and disc content. Nat. Rev. Rheumatol. 10, 44–56. 10.1038/nrrheum.2013.160 24166242 PMC4151534

[B61] Robinson M. D. McCarthy D. J. Smyth G. K. (2010). edgeR: a Bioconductor package for differential expression analysis of digital gene expression data. Bioinf. 26, 139–140. 10.1093/bioinformatics/btp616 PMC2796818 19910308

[B62] Roughley P. J. Alini M. Antoniou J. (2002). The role of proteoglycans in aging, degeneration and repair of the intervertebral disc. Biochem. Soc. Trans. 30, 869–874. 10.1042/bst0300869 12440935

[B63] Sadowska A. Touli E. Hitzl W. Greutert H. Ferguson S. J. Wuertz-Kozak K. (2017). Inflammaging in cervical and lumbar degenerated intervertebral discs: analysis of proinflammatory cytokine and TRP channel expression. Eur. Spine J. 27 (3), 564–577. 10.1007/s00586-017-5360-8 29204735 10.1007/s00586-017-5360-8

[B64] Sadowska A. Kameda T. Krupkova O. Wuertz-Kozak K. (2018). Osmosensing, osmosignalling and inflammation: how intervertebral disc cells respond to altered osmolarity. Eur. Cell Mater. 36, 231–250. 10.22203/eCM.v036a17 30452080

[B65] Sadowska A. Hitzl W. Karol A. Jaszczuk P. Cherif H. Haglund L. (2019). Differential regulation of TRP channel gene and protein expression by intervertebral disc degeneration and back pain. Sci. Rep. 9, 18889. 10.1038/s41598-019-55212-9 31827137 PMC6906425

[B66] Schmidt H. Shirazi-Adl A. Galbusera F. Wilke H. J. (2010). Response analysis of the lumbar spine during regular daily activities–a finite element analysis. J. Biomech. 43, 1849–1856. 10.1016/j.jbiomech.2010.03.035 20394933

[B67] Servin-Vences M. R. Moroni M. Lewin G. R. Poole K. (2017). Direct measurement of TRPV4 and PIEZO1 activity reveals multiple mechanotransduction pathways in chondrocytes. Elife 6, e21074. 10.7554/eLife.21074 28135189 PMC5279942

[B68] Souza Bomfim G. H. Costiniti V. Li Y. Idaghdour Y. Lacruz R. S. (2020). TRPM7 activation potentiates SOCE in enamel cells but requires ORAI. Cell Calcium 87, 102187. 10.1016/j.ceca.2020.102187 32146159 PMC7202080

[B69] Tashiro M. Inoue H. Konishi M. (2014). Physiological pathway of magnesium influx in rat ventricular myocytes. Biophys. J. 107, 2049–2058. 10.1016/j.bpj.2014.09.015 25418090 PMC4223180

[B70] Tashiro M. Inoue H. Konishi M. (2019). Modulation of Mg(2+) influx and cytoplasmic free Mg(2+) concentration in rat ventricular myocytes. J. Physiol. Sci. 69, 97–102. 10.1007/s12576-018-0625-5 29909547 PMC10717743

[B71] Thorneloe K. S. Sulpizio A. C. Lin Z. Figueroa D. J. Clouse A. K. McCafferty G. P. (2008). N-((1S)-1-{4-((2S)-2-{(2,4-dichlorophenyl)sulfonyl]amino}-3-hydroxypropanoyl)-1 -piperazinyl]carbonyl}-3-methylbutyl)-1-benzothiophene-2-carboxamide (GSK1016790A), a novel and potent transient receptor potential vanilloid 4 channel agonist induces urinary bladder contraction and hyperactivity: Part I. J. Pharmacol. Exp. Ther. 326 , 432–42. 10.1124/jpet.108.139295 18499743

[B72] Thorneloe K. S. Cheung M. Bao W. Alsaid H. Lenhard S. Jian M. Y. (2012). An orally active TRPV4 channel blocker prevents and resolves pulmonary edema induced by heart failure. Sci. Transl. Med. 4, 159ra148. 10.1126/scitranslmed.3004276 23136043

[B73] Ulger O. Demirel A. Oz M. Sahin A. (2018). Effectiveness of physiotherapy and minimal invasive technics on functional status and quality of life in geriatric patients with low back pain. J. Exerc. Rehabil. 14, 1048–1052. 10.12965/jer.1836354.177 30656168 PMC6323347

[B74] Walter B. A. Purmessur D. Moon A. Occhiogrosso J. Laudier D. M. Hecht A. C. (2016). Reduced tissue osmolarity increases TRPV4 expression and pro-inflammatory cytokines in intervertebral disc cells. Eur. Cells Mater. 32, 123–136. 10.22203/eCM.v032a08 PMC5072776 27434269

[B75] Weber K. T. Alipui D. O. Sison C. P. Bloom O. Quraishi S. Overby M. C. (2016). Serum levels of the proinflammatory cytokine interleukin-6 vary based on diagnoses in individuals with lumbar intervertebral disc diseases. Arthritis Res. Ther. 18, 3. 10.1186/s13075-015-0887-8 26743937 PMC4718017

[B76] Wieser S. Horisberger B. Schmidhauser S. Eisenring C. Brugger U. Ruckstuhl A. (2011). Cost of low back pain in Switzerland in 2005. Eur. J. Health Econ. 12, 455–467. 10.1007/s10198-010-0258-y 20526649 PMC3160551

[B77] Wilson C. Dryer S. E. (2014). A mutation in TRPC6 channels abolishes their activation by hypoosmotic stretch but does not affect activation by diacylglycerol or G protein signaling cascades. Am. J. Physiol. Renal Physiol. 306, F1018–F1025. 10.1152/ajprenal.00662.2013 24598806

[B78] Wuertz K. Urban J. P. Klasen J. Ignatius A. Wilke H. J. Claes L. (2007). Influence of extracellular osmolarity and mechanical stimulation on gene expression of intervertebral disc cells. J. Orthop. Res. 25, 1513–1522. 10.1002/jor.20436 17568421

[B79] Xu X. Gordon E. Lin Z. Lozinskaya I. M. Chen Y. Thorneloe K. S. (2009). Functional TRPV4 channels and an absence of capsaicin-evoked currents in freshly-isolated, guinea-pig urothelial cells. Channels (Austin) 3, 156–60. 10.4161/chan.3.3.8555 19411839

