# Supplementary material for: Hypo-Osmotic Loading Induces Expression of IL-6 in Nucleus Pulposus Cells of the Intervertebral Disc Independent of TRPV4 and TRPM7
Source: Front Pharmacol. 2020 Jul 1;11:952. doi: 10.3389/fphar.2020.00952 (PMC7341822; doi:10.3389/fphar.2020.00952)
Supplement: Supplementary file 1 [file DataSheet_1.docx]

Hypo-osmotic loading induces expression of IL-6 in nucleus pulposus cells of the intervertebral disc independent of TRPV4 and TRPM7

Aleksandra Sadowska^1^, Birsen Altinay^1^, Wolfgang Hitzl ^2,3,4^, Stephen J. Ferguson^1^, Karin Wuertz-Kozak^1,5,6*^

^1^Institute for Biomechanics, ETH Zurich, Zurich, Switzerland

^2^Research Office (Biostatistics), Paracelsus Medical University, Salzburg, Austria

^3^Paracelsus Medical University Salzburg, Department of Ophthalmology and Optometry Salzburg, Austria

^4^Research Program Experimental Ophthalmology and Glaucoma Research, Paracelsus Medical University, Salzburg, Austria

^5^Tissue Regeneration & Mechanobiology Lab, Department of Biomedical Engineering, Rochester Institute of Technology (RIT), Rochester, USA

^6^ Spine Center, Schön Clinic Munich Harlaching, Academic Teaching Hospital and Spine Research Institute of the Paracelsus Medical University Salzburg, Germany

*** Correspondence:**Dr. Karin Kozak-Wuertz
[kwbme@rit.edu](mailto:kwbme@rit.edu)

**Keywords:** Intervertebral disc (IVD), osmolarity, osmosensing, transient receptor potential (TRP) channels, inflammation, degenerative disc disease (DDD), membrane receptor, low back pain

**Supplementary Figure 1.** Dose response activation of TRPV4 with GSK1016790A in hypo-osmotic condition. Graphs present the ratio of the bound calcium to the unbound calcium (340 nm/380 nm) normalized to the baseline over the measurement time (mean ± SEM, bNP P2, n = 3). After the baseline measurement, empty or compound-supplemented media was added (indicated by an arrow on a graph) and the measurement was continued for up to around 15 min more. Asterisks indicates statistical significance (* p < 0.05, ** p ≤ 0.01, *** p ≤ 0.001, *ns*: no statistical difference defined as p > 0.05) as measured on the last measurement cycle.

**Supplementary Figure 2.** Dose response activation of TRPM7 with Naltriben methanesulfonate in hypo-osmotic condition. Graphs present the ratio of the bound calcium to the unbound calcium (340 nm/380 nm) normalized to the baseline over the measurement time (mean ± SEM, bNP P2, n = 3). After the baseline measurement, empty or compound-supplemented media was added (indicated by an arrow on a graph) and the measurement was continued for up to around 15 min more. Asterisks indicates statistical significance (* p < 0.05, ** p ≤ 0.01, *** p ≤ 0.001, *ns*: no statistical difference defined as p > 0.05) as measured on the last measurement cycle.

**Supplementary Figure 3.** Ca^2+^ flux in NP cells following the application of 40 nM GSK1016790A (TRPV4 activator) in hypo- and iso-osmotic condition. Graphs present the ratio of the bound calcium to the unbound calcium (340 nm/380 nm) normalized to the baseline over the measurement time (mean ± SEM, bNP P2, n = 3). After the baseline measurement, empty (control) or compound-supplemented (treatment) media was added (indicated by an arrow on a graph) and the measurement was continued for up to around 15 min more. Asterisks indicates statistical significance (* p < 0.05, ** p < 0.01, *** p < 0.001, *ns*: no statistical difference defined as p > 0.05) as measured on the last measurement cycle.
